# Partner Notification for Reduction of HIV-1 Transmission and Related Costs among Men Who Have Sex with Men: A Mathematical Modeling Study

**DOI:** 10.1371/journal.pone.0142576

**Published:** 2015-11-10

**Authors:** Brooke E. Nichols, Hannelore M. Götz, Eric C. M. van Gorp, Annelies Verbon, Casper Rokx, Charles A. B. Boucher, David A. M. C. van de Vijver

**Affiliations:** 1 Department of Viroscience, Erasmus Medical Center, Rotterdam, the Netherlands; 2 Department Infectious Disease Control, Public Health Service Rotterdam-Rijnmond, Rotterdam, the Netherlands; 3 Department of Public Health, Erasmus Medical Center, Rotterdam, the Netherlands; 4 Department of Internal Medicine and Infectious Diseases, Erasmus Medical Center, Rotterdam, the Netherlands; Centers for Disease Control and Prevention, UNITED STATES

## Abstract

**Background:**

Earlier antiretroviral treatment initiation prevents new HIV infections. A key problem in HIV prevention and care is the high number of patients diagnosed late, as these undiagnosed patients can continue forward HIV transmission. We modeled the impact on the Dutch men-who-have-sex-with-men (MSM) HIV epidemic and cost-effectiveness of an existing partner notification process for earlier identification of HIV-infected individuals to reduce HIV transmission.

**Methods:**

Reduction in new infections and cost-effectiveness ratios were obtained for the use of partner notification to identify 5% of all new diagnoses (Scenario 1) and 20% of all new diagnoses (Scenario 2), versus no partner notification. Costs and quality adjusted life years (QALYs) were assigned to each disease state and calculated over 5 year increments for a 20 year period.

**Results:**

Partner notification is predicted to avert 18–69 infections (interquartile range [IQR] 13–24; 51–93) over the course of 5 years countrywide to 221–830 (IQR 140–299; 530–1,127) over 20 years for Scenario 1 and 2 respectively. Partner notification was considered cost-effective in the short term, with increasing cost-effectiveness over time: from €41,476 -€41, 736 (IQR €40,529-€42,147; €40,791-€42,397) to €5,773 -€5,887 (€5,134-€7,196; €5,411-€6,552) per QALY gained over a 5 and 20 year period, respectively. The full monetary benefits of partner notification by preventing new HIV infections become more apparent over time.

**Conclusions:**

Partner notification will not lead to the end of the HIV epidemic, but will prevent new infections and be increasingly cost-effectiveness over time.

## Introduction

In 2012, the HIV epidemic among men who have sex with men (MSM) was increasing across the world [1, 2]. MSM are disproportionately affected by HIV infection in the Netherlands comprising 71% of new HIV-infections in 2013 [3]. Next to behavior change and condom use, the use of treatment as prevention has shown to be an effective way to prevent HIV [4]. Among men and women in a generalized epidemic, the initiation of antiretroviral therapy (ART) at a CD4 cell count between 350–550 cells/μl was shown to prevent 96% of new infections as compared to treatment initiation at CD4 <250 cells/μl [4, 5]. It is hypothesized that the effect of treatment as prevention is similar among MSM [6].

Treatment as prevention cannot succeed unless patients are diagnosed early in infection. Early identification of patients is key from both clinical and preventative perspectives. Patients who are identified early and initiate antiretroviral therapy at a higher CD4 cell count have lower mortality, and fewer long-term complications and opportunistic infections compared to patients who initiate treatment late [4, 7]. Unfortunately, a significant problem in HIV prevention and care is the substantial number of patients diagnosed late.[8] Across Europe, MSM are diagnosed late in infection, with 44% diagnosed at a CD4 count <350, and 24% diagnosed with a CD4 <200 cells/μl [8]. The number of late diagnoses is similar among MSM in the Netherlands, with 37% of individuals diagnosed with a CD4 count <350 cells/μl, of which approximately half are diagnosed with CD4 <200 cells/μl [3]. These undiagnosed patients can then continue the forward transmission of HIV-1. It has been estimated that 50% of new infections are due to the 20% who are unaware of their infection [9], though these percentages may vary by setting.

Partner notification can play a role in identifying a proportion of HIV-infected individuals who are unaware of their infection, and getting them into care earlier in infection [10]. Notified partners have a higher rate of HIV positivity than those who come in for screening without being notified [10–13]. If HIV is diagnosed in a notified partner, these individuals can then initiate treatment earlier which can in turn reduce the number of HIV infections to others. Partner notification is therefore a method that can be used to control sexually transmitted infections (STIs) and HIV [14]. The cost-effectiveness and full monetary benefit of partner notification is not yet known.

The aim of this study is to use mathematical modeling to determine the preventative impact on new HIV-1 infections and cost-effectiveness of partner notification. For this purpose, we used data from the Rotterdam-Rijnmond Public Health Service (the Netherlands) which utilizes partner notification supported by an online partner notification system.

## Methods

### Study design and partner notification

We based our model on the outcomes of partner notification supported by an online partner notification tool in the Rotterdam region. The online tool, known as Suggest-A-Test, has been implemented by both the Rotterdam-Rijnmond and Amsterdam health municipalities since 2012 [10]. Suggest-A-Test is a tool in which people diagnosed with an STI/HIV can easily and anonymously notify recent partners. After a patient is diagnosed, there is an intensive counseling process at the STI clinic in which partner notification is discussed. Patients can choose whether to contact their partners on their own or through the Suggest-A-Test system, and most choose to notify partners outside the Suggest-A-Test tool. For an HIV diagnosis, it is advised that the patient notifies all partners from the last 12 months and longer if possible.

We modeled two partner notification scenarios using the partner notification outcome data. In 2013, there were nine new HIV diagnoses via partner notification out of 366 MSM notified for any STI/HIV and tested for HIV. These nine new diagnoses represent approximately 4·7% of all new diagnoses in the entire Rotterdam region. Therefore in Scenario 1, we assume that approximately 5% of diagnoses in the Netherlands can be ascertained through partner notification. The nine new diagnoses also represent 19·6% of new diagnoses at the Rotterdam Public Health Municipality. We then model this in Scenario 2, in which we assume approximately 20% of individuals are diagnosed through partner notification. Scenario 1 therefore represents a decrease in effectiveness that may be observed with a nationwide scale-up of partner notification.

### Model assumptions and calibration

A compartmental deterministic mathematical model was constructed and parameters were chosen to represent the Dutch HIV epidemic among MSM from 2008–2012 (Table 1) [15, 16]. We estimated that there were approximately 176,000 (164,000–190,000) MSM in the Netherlands in 2014 over the age of 15 [17, 18], with the number of new HIV diagnoses declining from 800 in 2008 to 700 in 2012 among MSM [3]. We model the partner notification process when treatment is initiated at CD4 cell count of <500 cells/μl, in line with current World Health Organization guidelines [19], and when treatment is initiated immediately, in line with current Dutch guidelines [20]. Partner notification is implemented in 2015, and the model is run until 2035. We compare our partner notification scenarios with treatment at CD4 <500 cells/μl, with a baseline of no partner notification and treatment at CD4 <500 cells/μl; and similarly our partner notification scenarios with immediate treatment, with a baseline of no partner notification and immediate treatment.

**Table 1:** Key model parameters and costs.

Description	Estimate or Range*	Reference
Model parameters		
*Disease stages duration*		[21, 22]
Acute stage	10–16 weeks	
Chronic stage >500 cells/μL	0.87–1 year	
Chronic stage 350–500 cells/μL	2·9–3·1 years	
Chronic stage 200–350 cells/μL	3·6–3·9 years	
AIDS stage**	6–12 months	
Final AIDS stage**	7–13 months	
*Infectivity; per partnership transmissibility per year*		[23]; Model Calibration
Acute stage	0·024–0·59%	
Chronic stage (all)	0·023–0·22%	
AIDS stage**	0·006–0·27%	
Final AIDS stage**	0%	
*Proportion of people in sexual risk groups*		Model Calibration
Highest	1·0–3·8%	
2^nd^	11–40%	
3^rd^	10–60%	
Lowest	12–70%	
*Number of partners per year in each sexual risk group*		Model Calibration
Highest	92–556	
2^nd^	10–91	
3^rd^	1–9·9	
Lowest	0·4–2	
*Mortality rates per year*		[24–26]
Population	0·0155	
Chronic HIV stage	0·098	
AIDS stage	0·63	
On treatment during chronic stage, first 3 months	0·0172–0·0175	
On treatment during AIDS stage, first 3 months	0·0184–0·0196	
On treatment 3+ months	0·0172–0·0175	
*HIV Test Rate*		
Baseline	15·5–20%	Model Calibration
Rate of being tested in the acute stage of HIV	80–87·5% of the baseline rate	Assumption***
Linkage to care from test to treat	90–98%	Model Calibration
Reduction in transmissibility of those patients on treatment	90–100%	[4, 6, 27]
**Cost Parameters (Costs listed are in 2015 euros)**		
*Testing*		
Primary HIV test^†^	€20·32	Local data
Confirmatory testing	€45·83	Local data
All-inclusive cost charged to STI clinic for all STI tests (chlamydia, gonorrhea, syphilis, hepatitis B and HIV combined)	€124	Local data
*ART costs*		
Yearly cost of ART (averaged across regimens by number of people on regimen)	€10293	Local data
CD4 cell count test	€97·75	Local data
Viral load test	€66·54	Local data
Outpatient visit at clinic/primary care	€31	[28]
Outpatient visit after diagnosis^‡^	€124	[28], Local data
Outpatient visit for partner notification^‡^	€124	[28], Local data
Outpatient visit at HIV specialist	€143	[28]
*Cost of treating opportunistic infections* ^§^		Local data
Recent infection	€4·47-€21·62	
CD4 350–500	€9·26-€48·61	
CD4 200–350	€40·65-€76·87	
CD4 50–200	€104·63-€476·15	
CD4 <50	€403·26-€721·93	
First 3 months on treatment	€10·81-€238·08	

*All ranges are uniformly distributed

**Two AIDS stages were included because during the final months before death, patients have limited sexual activity

*** Due to window phase of p24 antigen testing

† Includes cost of false positives that require additional testing (0·7% false positivity rate)

‡ Four times the length of a normal outpatient clinic appointment

^§^ The average cost per person per stage of infection/treatment. Includes diagnosis, treatment, personnel costs. Averaged per patient per year.

Our model stratifies disease progression into an acute stage, three chronic stages, and two AIDS stages (the full model schematic can be found in S1 Fig). Three chronic stages were chosen to be able to evaluate the model when treatment is initiated at different CD4 cell count thresholds: CD4 cell count <200, <350, <500 cells/μl, and immediate treatment. The duration and infectivity of each stage of infection differ (Table 1) [22, 23]. Treatment is assumed to reduce infectivity by 90–100% [4, 6]. In the model, approximately 25–30% of patients are diagnosed with a CD4 cell count >500 cells/μl, and approximately 35–40% diagnosed with a CD4 cell count <350 cells/μl, in line with current data[3]. In our baseline scenarios with no partner notification, we assume that individuals are tested at the rates that allow the modelled CD4 cell count distribution at diagnosis to match the current CD4 cell count distribution at diagnosis in the Dutch HIV epidemic among MSM. As the CD4 cell count at diagnosis of notified partners follows approximately the same distribution as those who were tested without being notified, we increased the test rate at all stages of infection in our partner notification scenarios. Full model equations and description can be found in S1 Text of the supporting information.

We matched our model to the previous epidemic based on: estimated Dutch MSM population size, number diagnosed with HIV, percentage diagnosed with a CD4 <200 cells/μl, and percentage diagnosed with CD4 200–350 cells/μl. Using Monte Carlo filtering techniques [29], we accepted 129 of 100,000 simulations that matched these parameters (value ranges of accepted parameters can be found in S1 Table). The model calibration to the number diagnosed is shown in S2 Fig. All reported results are the median and interquartile range (IQR) of the accepted simulations.

### Cost-effectiveness of partner notification

Each compartment in our deterministic model was assigned a cost and quality adjusted life year (QALY) depending on the intervention (Table 1, QALY weights can be found in S2 Table). In this analysis we take a third-party-payer perspective, and as such we take local costs for hospitalization of HIV infected persons, opportunistic infections, HIV testing, and ART, into account. We calculated incremental cost-effectiveness ratios over a 5, 10, 15, and 20 year period where we compared incremental costs and QALYs of partner notification scenarios to the baseline of no partner notification by treatment initiation threshold. Costs were discounted at 4% per year, and QALYs at 1·5% per year, as per Dutch guidelines [30].

### Sensitivity analysis

We performed a univariate sensitivity analysis of the cost-effectiveness of partner notification of Scenario 1- identifying approximately 5% of new HIV cases via partner notification. Five key input variables–number needed to test via partner notification for a positive HIV diagnosis, cost of antiretroviral drugs, cost of HIV testing, cost discounting, and QALY discounting—were considered to identify the sensitivity of our model. Recursive partitioning [31, 32] was conducted to determine the most influential parameters on the number of infections averted when using partner notification (S3 Fig shows the recursive partitioning analysis).

## Results

### Impact on Dutch HIV epidemic

#### Scenario 1

**Fig 1:**
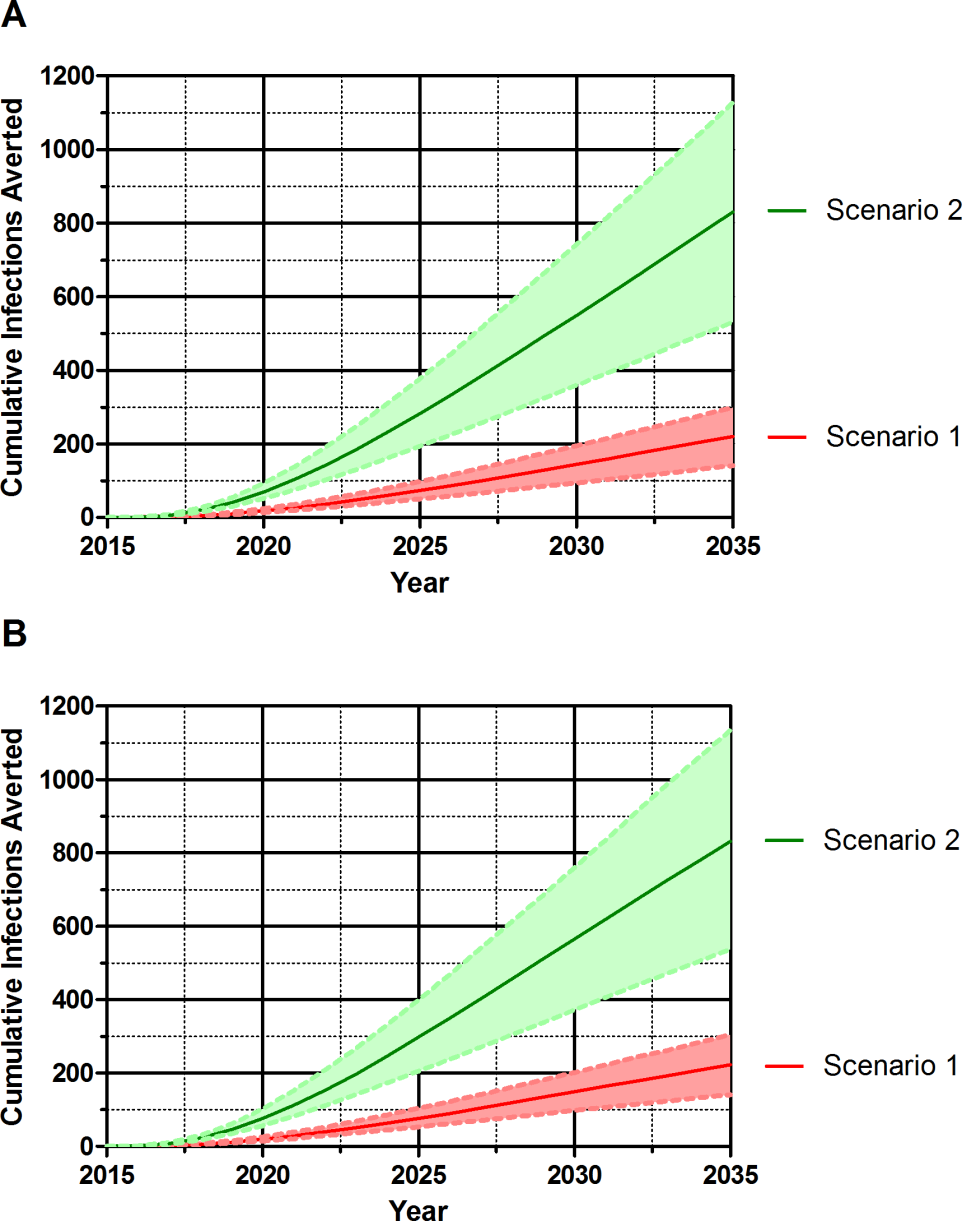
Cumulative infections averted over 20 years. Scenario 1 in which 5% of patients are diagnosed through the online partner notification system (in red, median and interquartile range). Scenario 2 in which 20% of patients are diagnosed through the online partner notification system (in green, median and interquartile range). Graph A is when treatment is initiated at a CD4 cell count <500 cells/μl. Graph B is when treatment is initiated immediately.

When 5% of new infections are identified through partner notification, partner notification is predicted to avert a total of 18 and 19 infections (interquartile range [IQR] 13–24; 14–26) over the course of 5 years countrywide when initiating at CD4 <500 cells/μl and immediately, respectively compared to treatment at those two thresholds with no partner notification use. Over 20 years, partner notification is predicted to avert between 221 and 222 infections (IQR 140–299; 140–304) (Fig 1) when initiating at CD4 <500 cells/μl and immediately, respectively. This represents approximately 1.5% of new infections over the 20 year timeframe.

#### Scenario 2

When 20% of new infections are identified via partner notification, on average 69 and 76 infections (IQR 51–93; 56–102) are predicted to be averted over 5 years. Over 20 years, between 830 and 832 infections (IQR 530–1,127; 537–1,135) are predicted to be averted when initiating at CD4 <500 cells/μl and immediately, respectively. Averting nearly four times more infections than Scenario 1, the number of infections averted represents approximately 5.7% of new infections over 20 years.

### Cost-effectiveness of partner notification

**Fig 2:**
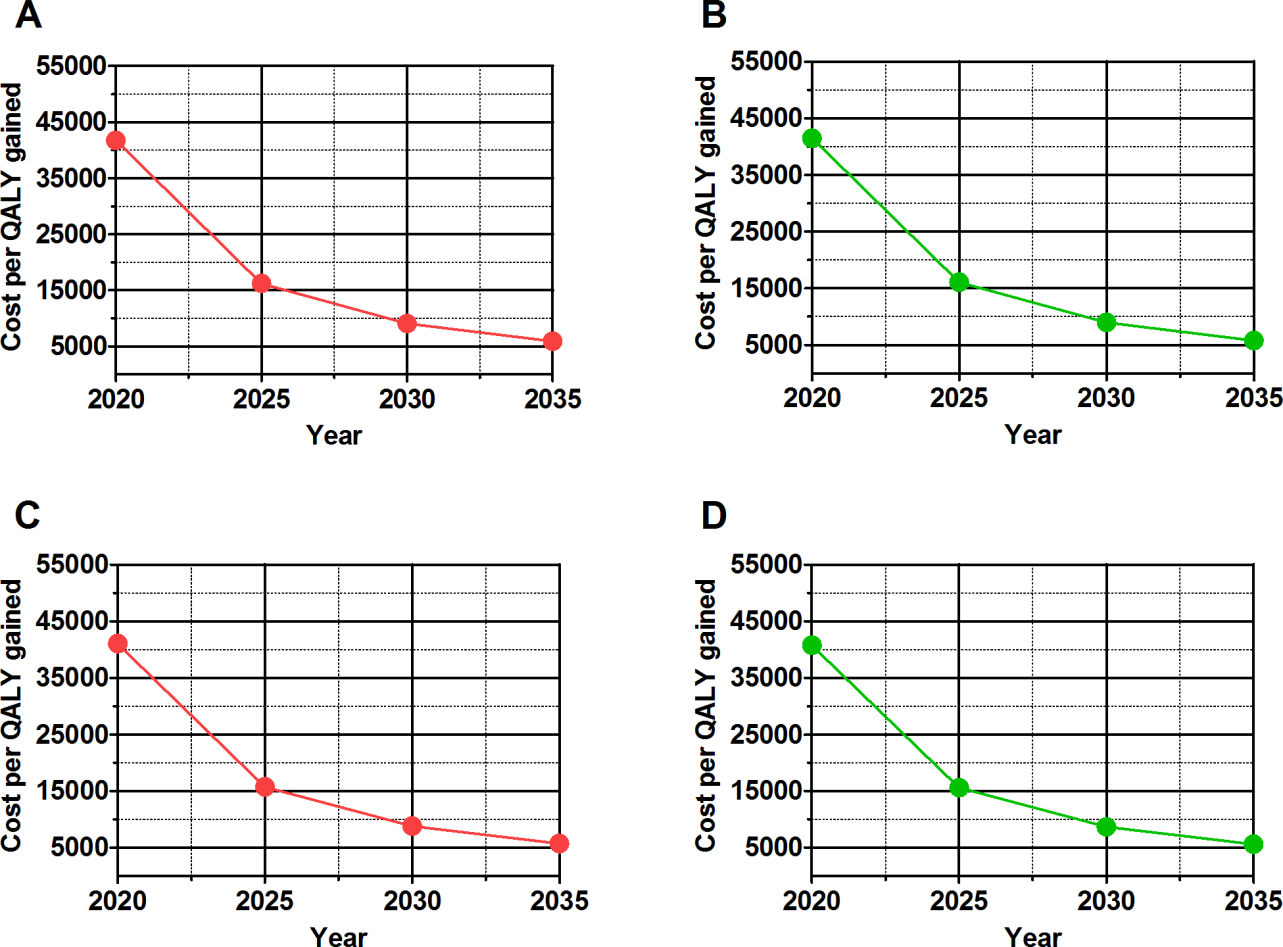
Cost per quality adjusted life year (QALY) gained over time in 5 year increments. Scenario 1 in which 5% of patients are diagnosed through partner notification (Graph A when treatment is started a CD4 cell count <500 cells/μl, Graph C when treatment is initiated immediately). Scenario 2 in which 20% of patients are diagnosed through partner notification. Graph A is when treatment is initiated at a CD4 cell count <500 cells/μl. Graph B is when treatment is initiated immediately (Graph B when treatment is started a CD4 cell count <500 cells/μl, Graph D when treatment is initiated immediately).

Partner notification had substantial increasing cost-effectiveness over time: from €41,736 (IQR €40,791-€42,397) per QALY gained over 5 years to €5,887 (€5,411-€6,552) per QALY gained over 20 years in Scenario 1 when treatment is initiated at CD4 <500 cells/µl (Table 2). When treatment is initiated immediately, the cost per QALY decreases slightly to €41,065 (IQR €39,261-€42,134) per QALY gained at 5 years and €5,719 (IQR €5,113-€6,339) per QALY gained at 20 years in Scenario 1. The cost-effectiveness ratios for Scenario 2, where 20% of HIV diagnoses are through partner notification, are only reduced by 1–2% (Table 2, Fig 2). The full monetary benefits of partner notification by preventing new HIV infections become more apparent over time.

**Table 2:** Incremental cost-effectiveness of partner notification (Scenario 1 in which 5% are diagnosed via online partner notification, and Scenario 2 in which 20% of patients are diagnosed via online partner notification, and worst case scenario): at 5, 10, 15 and 20 years. All values listed are the median of all model simulations and interquartile range of simulations.

Intervention	Total Cost (Millions Euros)	QALYs Gained	Infections Averted	Incremental Cost-Effectiveness Ratio
**Treat at CD4 cell count <500 cells/μl**				
**5 years**				
Scenario 1*	€1,644,473 (€1,322,401-€1,977,121)	39 (31–50)	18 (13–24)	€41,736 (€40,791-€42,397)
Scenario 2**	€6,433,290 (€5,175,973-€7,731,741)	155 (122–196)	69 (51–93)	€41,476 (€40,529-€42,147)
**10 years**				
Scenario 1*	€4,264,124 (€3,451,544-€5,250,083)	262 (204–335)	73 (50–98)	€16,193 (€15,627-€16,926)
Scenario 2**	€16,376,219 (€13,289,756-€20,168,533)	1,019 (792–1,300)	282 (193–377)	€16,044 (€15,456-€16,784)
**15 years**				
Scenario 1*	€6,580,279 (€5,276,477-€8,335,685)	756 (553–941)	144 (94–195)	€9,057 (€8,561-€9,777)
Scenario 2**	€25,114,652 (€20,123,588-€31,731,638)	2,903 (2,126–3,612)	549 (359–741)	€8,944 (€8,454-€9,679)
**20 years**				
Scenario 1*	€8,499,662 (€6,783,954-€10,817,655)	1,519 (1,081–1,890)	221 (140–299)	€5,887 (€5,411-€6,552)
Scenario 2**	€32,005,785 (€25,472,626-€40,567,125)	5,773 (4,134–7,196)	830 (530–1,127)	€5,773 (€4,134-€7,196)
**Immediate Treatment**				
**5 years**		-	-	-
Scenario 1*	€1,713,341 (€1,387,291-€2,114,998)	41 (34–54)	19 (14–26)	€41,065 (€39,261-€42,134)
Scenario 2**	€6,727,578 (€5,407,791-€8,101,635)	165 (131–210)	76 (56–102)	€40,739 (€39,659-€41,521)
**10 years**				
Scenario 1*	€4,327,315 (€3,515,059-€5,287,711)	275 (214–359)	76 (53–104)	€15,735 (€15,155-€16,551)
Scenario 2**	€16,511,947 (€13,510,976-€20,413,643)	1,063 (828–1,365)	298 (204–399)	€15,595 (€15,074-€16,364)
**15 years**				
Scenario 1*	€6,603,666 (€5,258,305-€8,243,750)	757 (569–979)	149 (97–201)	€8,793 (€8,221-€9,450)
Scenario 2**	€25,140,754 (€20,032,146-€31,254,688)	2,993 (2,190–3,721)	565 (371–760)	€8,663 (€8,092-€9,381)
**20 years**				
Scenario 1*	€8,363,538 (€6,582,787-€10,501,999)	1,517 (1,120–1,939)	222 (140–304)	€5,719 (€5,113-€6,339)
Scenario 2**	€31,372,511 (€24,810,117-€39,483,106)	5,830 (4,244–7,291)	832 (537–1,135)	€5,616 (€5,028-€6,266)

*5% of patients diagnosed via partner notification

**20% of patients diagnosed via partner notification

### Sensitivity analysis

**Fig 3:**
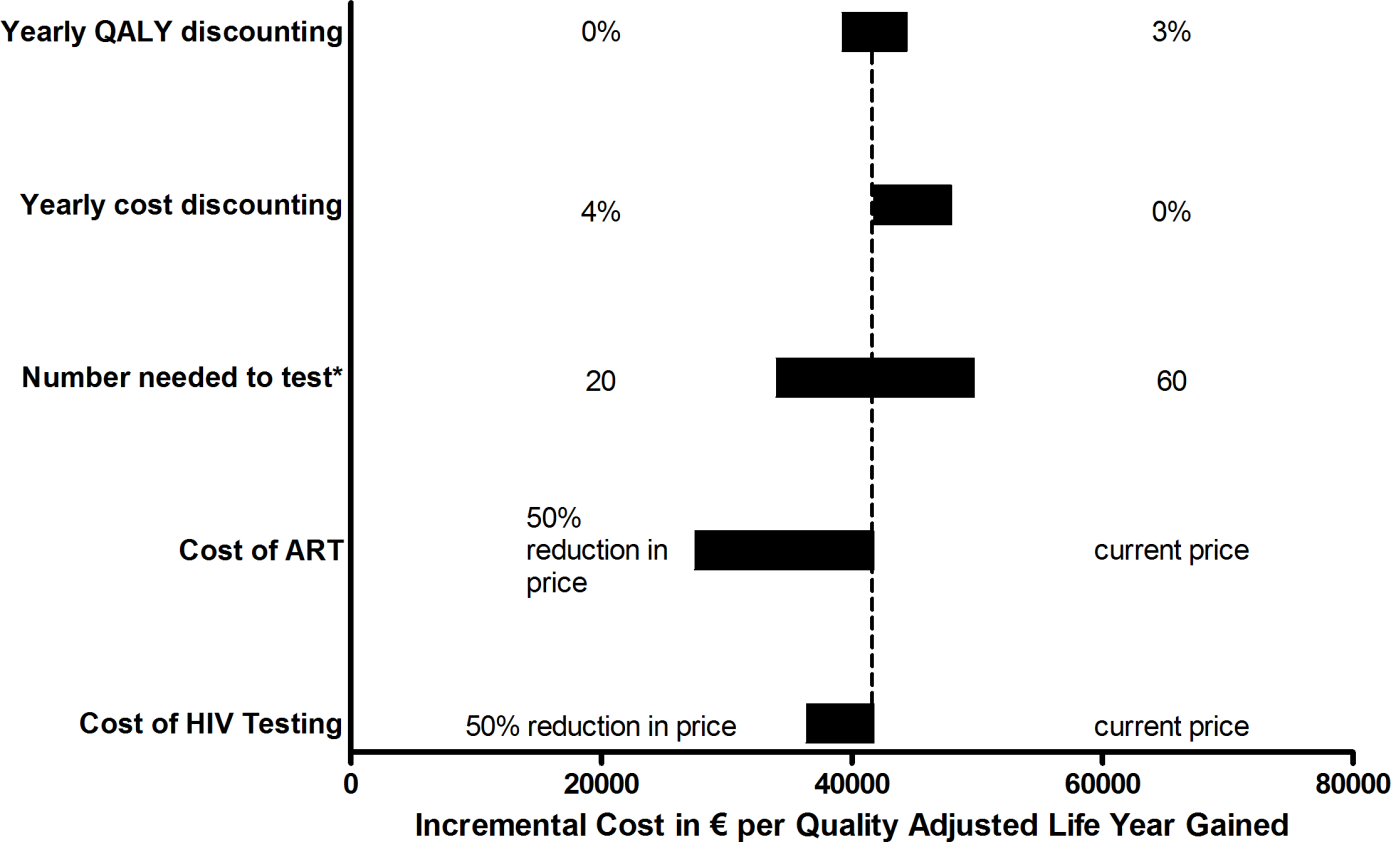
One-way sensitivity analyses of the incremental cost-effectiveness ratio of using the partner notification system over 5 years in Scenario 1 in which 5% of patients are diagnosed via the online partner notification system. Each horizontal bar represents the full range of cost-effectiveness ratios produced by varying a given model parameter across its plausible range. The vertical dotted line represents the incremental cost-effectiveness ratio in Scenario 1 over 5 years in the primary analysis (€41,736 per QALY gained).

One-way sensitivity analyses (Fig 3) highlighted the five key input parameters of our model. If the yearly cost discounting is assumed to be 0%, or the number needed to test increases to 60 per 1 HIV diagnosis, then the cost per QALY gained of partner notification increases to €47,953 (IQR €46,833-€48,680) and €49,727 (IQR €48,606-€50,585) respectively. Yearly QALY discounting reduced to 0%, the number needed to test decreases to 20 per 1 HIV diagnosis, or the cost of ART or HIV testing is decreased by 50%, partner notification becomes even more cost-effective. Decreasing the cost of ART is the parameter that results in the largest change in cost per QALY, with a median decrease of 34.2% in cost per QALY gained to €27,447 (IQR €26,864-€27,941).

## Discussion

Partner notification supported by an online partner notification tool is an approach to get individuals at high risk for HIV transmission to test for HIV. Partner notification was increasingly cost-effective over time, even in Scenario 1 in which only 5% of patients are diagnosed through partner notification. More infections could be diagnosed in a timely manner if partner notification is improved. Identifying patients earlier is more likely to reduce the epidemic as the acute stage has a higher infectivity[33]. If acutely infected patients are identified and notify their partners who may also have been acutely infected, whole transmission clusters could be prevented. Not only can patients who test earlier initiate treatment earlier, but they may also reduce their risk behavior, preventing additional infections [34]. Therefore, ways in which to prioritize and improve HIV partner notification to get patients to test earlier, with a focus on recent infections, should be explored.

In this study, we have modeled the effectiveness of an existing partner notification process. As such, the utilized data includes all of the pitfalls of implementing this process in practice. While the efficacy of partner notification could be higher, we have chosen to model this real-world scenario. We have shown, however, that even if the process becomes more effective and the number needed to test to diagnose one HIV patient decreases to 20, the cost-effectiveness ratio only decreases by 21%. As shown in our sensitivity analysis, decreasing the price of antiretroviral drugs can be even more successful at reducing the cost per QALY gained. This is because partner notification gets patients into care sooner, and therefore potential earlier initiation of costly ART. In the Netherlands, tenofovir-containing drugs, including combination tenofovir-emtricitabine and combination tenofovir-emtricitabine-efavirenz, were the 2^nd^ and 3^rd^ most expensive outpatient drug per patient in 2013 [35]. As generics come onto the market, ART price reduction will become a straightforward method of reducing costs for partner notification [36]. Costs due to additional HIV testing and counseling may be overestimated as notified individuals may eventually have accessed testing without being notified. Our analysis is therefore a worst-case scenario in which we assume the HIV tests and counselling resulting from partner notification are all additional costs. This overestimation of costs may be offset slightly by the initial unknown costs of a partner notification program, such as personnel training.

The effectiveness of the modeled partner notification is similar to other partner notification systems among MSM in similar settings [37]. Some studies have, however, shown a greater effectiveness of partner notification in identifying previously unknown HIV-infected persons in concentrated epidemics [12, 13, 38] As we have shown in our sensitivity analysis, an increased effectiveness would only make partner notification more cost-effective.

Many mathematical modeling studies have been performed in recent years that investigate the preventative effect of earlier ART initiation in resource rich settings [23, 39–43]. Models agree that earlier treatment initiation can reduce costs and will avert incident HIV infections over time. Studies have yet to explicitly model how to logistically get patients to test earlier, and just one model was based on a European MSM epidemic [43]. Our study addresses this by modeling a method that can get patients to test earlier. We find, however, a relatively limited impact of partner notification given the fact that a large proportion of sexual contacts are anonymous and cannot be notified [44]. Our model adds to the previous literature on mathematical modelling of partner notification by of the addition of dynamic HIV transmission and parameterization to real partner notification data within the model[45]. While modeling very different settings and partner notification programs and including dynamic HIV transmission directly into our model, we find a nearly identical cost per infection averted between our long-term analysis and the primary analysis conducted by Varghese et al. ($32,000 per infection averted in Varghese et al., and approximately €38,000 per infection averted in our 20 year analysis) [45].

Other ways to get patients into care early should be further investigated and modeled. Mobile testing units have had successes in a broad variety of settings [46–49], and is currently implemented in the Rotterdam region. Increasing awareness among general practitioners, along with physicians from other specialties, and efforts to normalize HIV testing can also be of importance, particular in resource-rich settings with low general HIV prevalence [50–52]. Many patients who were diagnosed late in infection had visited their general practitioner in the years before diagnosis with symptoms that could suggest an HIV infection. General practitioners that do more frequent HIV testing, especially among known at-risk populations such as MSM, can help to identify HIV. As such, increased HIV testing by general practitioners can lead to a reduction in the number of infected individuals who are diagnosed late [51, 52]. Finally, it has been shown that a large proportion of high sexual risk behavior MSM do not get tested regularly [53]. Therefore, efforts to increase awareness and testing among high-risk MSM may be a cost-effective strategy to get patients tested and into care earlier in infection.

Our mathematical model has several strengths. First, to our knowledge this is the first study to model and predict the long-term effectiveness of HIV partner notification. While previous models have focused on the effect of getting people into care sooner [23, 39–43], models have not been created to determine how to implement this. Second, we have access to complete and accurate data of the Dutch HIV epidemic and were able to successfully calibrate our model to that data. Finally, we modeled the effectiveness of a program using real programmatic data. This allows us to make accurate and practical predictions on the effectiveness of the partner notification process.

This study has some potential limitations. First, it is unknown what proportion of new infections can be diagnosed through partner notification when scaled up. To address this we have looked at both 5% and 20% of new diagnoses being identified via partner notification. While the preventative impact of partner notification was predicted to be higher if more individuals come in through partner notification, the cost-effectiveness is virtually identical regardless of the percentage of patients identified through partner notification. Second, there are limitations of the partner notification process itself. Just 46% of the partners of HIV-infected MSM were identifiable [44]. Of the partners that were identifiable however, nearly all were notified [44]. The partner notification in the Netherlands was shown to be similarly effective as a comparable process in the United States [44, 54]. While notifying anonymous partners appears difficult, it may be possible to notify a network of people that may have had contact with an infected individual, i.e. contacting individuals that visit the same sex club or dating website [55]. Cost-effectiveness may be increased and additional infections could be averted if these other techniques are implemented simultaneously. We have chosen to model the existing partner notification process, as predictions based on real data can be made with greater confidence. Third, we did not model a change in risk behavior after an HIV diagnosis or after ART initiation, as we did not have reliable data on this for our setting and we wanted to model a worst-case scenario. Other research shows that risk behavior can decrease after a positive test and ART initiation [34, 56]. If we had included a decrease in risk behavior in our model, we would expect the preventative impact of partner notification to be larger, and that partner notification would be more cost-effective. Finally given the ongoing and unresolved debate surrounding cost-effectiveness thresholds, we have chosen to not compare our costs per QALY gained to a threshold in this analysis [57, 58].

Not only can infections be averted using partner notification, but there is additional clinical and monetary benefit of the early identification of HIV in the short and long-term. Thus, while partner notification will not lead to end of the HIV epidemic, it does prevent new infections and have increasing cost-effectiveness over time. As such, it should be considered for implementation throughout the Netherlands and countries with similar epidemics.

## Supporting Information

S1 Fig Model schematic. (DOCX)

S2 Fig Calibration to number of newly diagnosed MSM patients: modeled data in green (median and interquartile range), and Dutch data in purple. (DOCX)

S3 Fig Recursive partitioning analysis. Higher epsilon value (greater than 0.61) was the strongest predictor for a reduction in new infections. A higher epsilon value means a higher rate of assortative mixing, or people who are highly sexually active are more likely to have sex with people who also are highly sexually active. The next strongest predictor for a reduction in new infections, among those simulations with a high epsilon value, is the number of HIV diagnosed among MSM in 2012. The simulations that had >730 new diagnoses also had the largest number of infections averted.
(DOCX)

S1 Table Variables used to calibrate and accept simulations using the Monte Carlo filtering technique. (DOCX)

S2 Table Assumed utility weightings for QALYs. (DOCX)

S1 Text Full model description and equations. (DOCX)
