# Supplementary figures and images for: Partner Notification for Reduction of HIV-1 Transmission and Related Costs among Men Who Have Sex with Men: A Mathematical Modeling Study
Source: PLoS One. 2015 Nov 10;10(11):e0142576. doi: 10.1371/journal.pone.0142576 (PMC4640527; doi:10.1371/journal.pone.0142576)

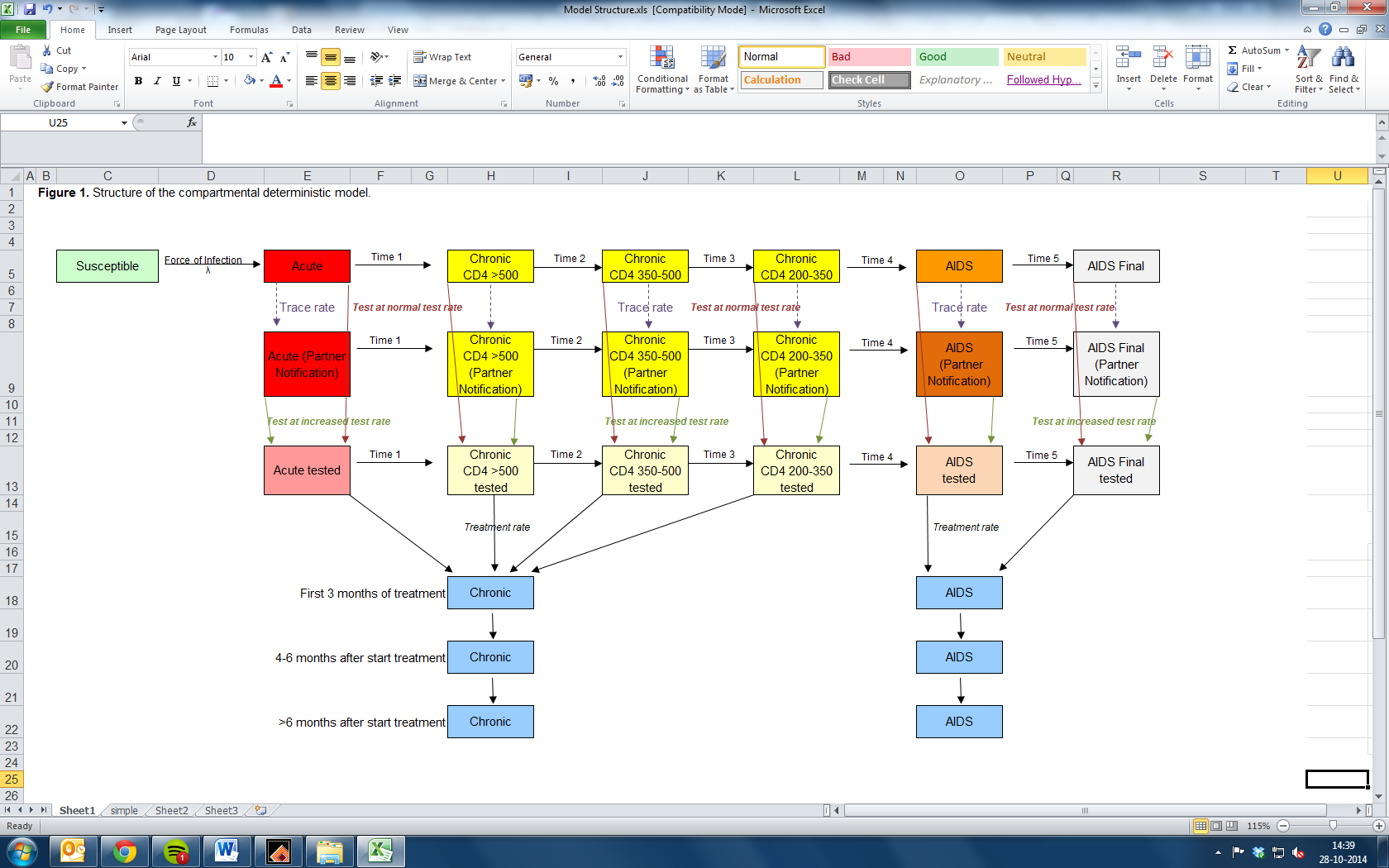

Supplement: S1 Fig — (DOCX) [file pone.0142576.s001.docx]

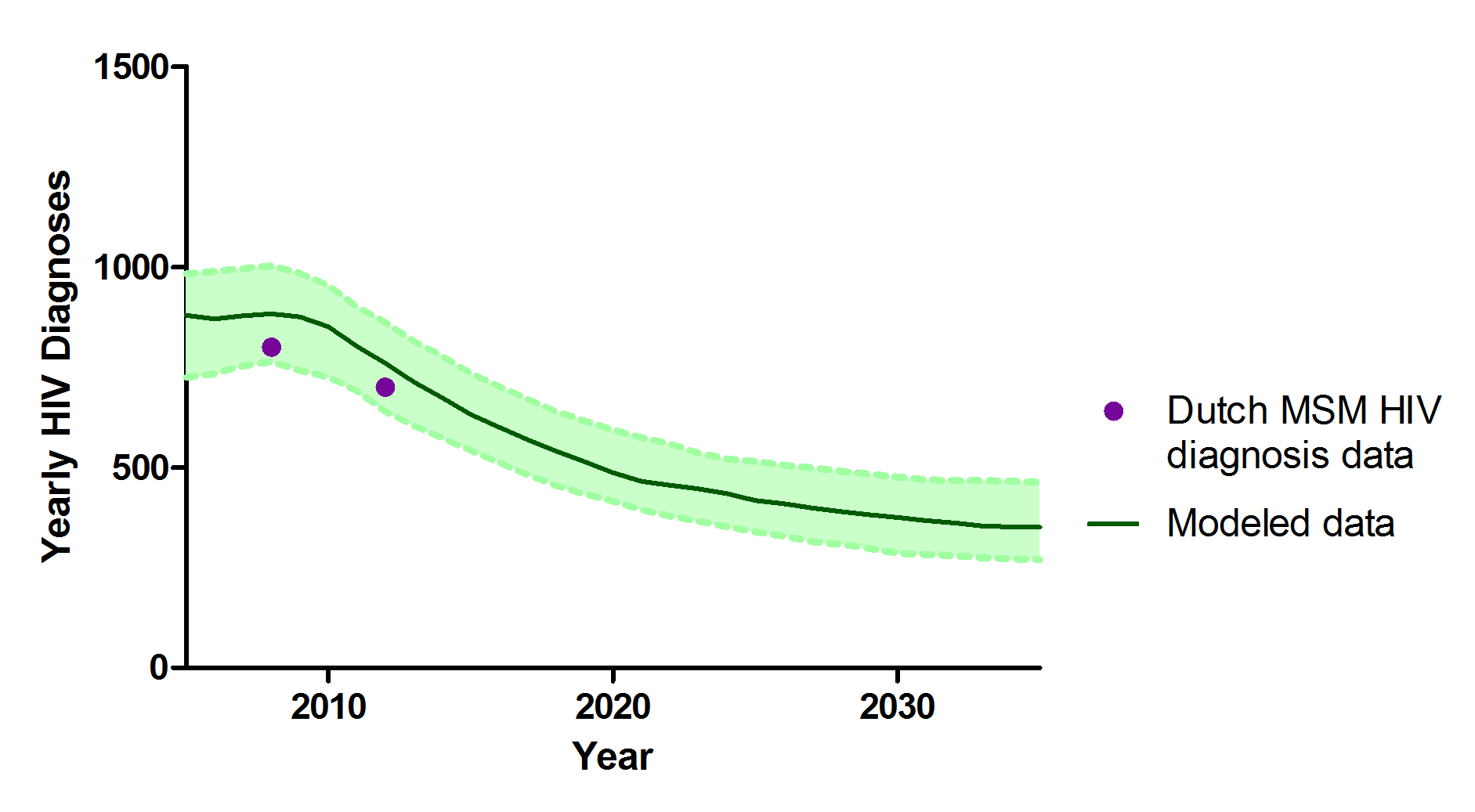

Supplement: S2 Fig — (DOCX) [file pone.0142576.s002.docx]

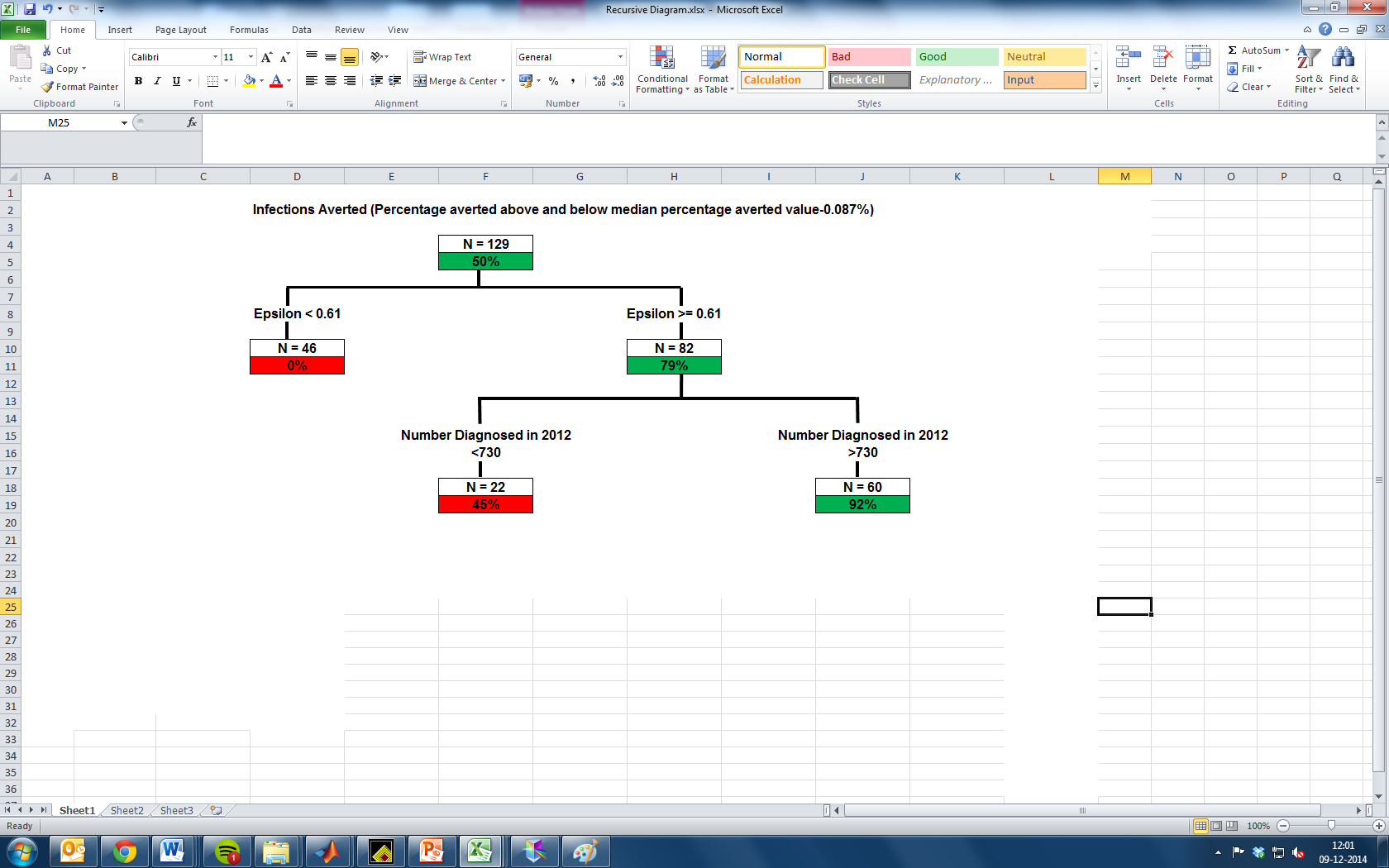

Supplement: S3 Fig — Higher epsilon value (greater than 0.61) was the strongest predictor for a reduction in new infections. A higher epsilon value means a higher rate of assortative mixing, or people who are highly sexually active are more likely to have sex with people who also are highly sexually active. The next strongest predictor for a reduction in new infections, among those simulations with a high epsilon value, is the number of HIV diagnosed among MSM in 2012. The simulations that had >730 new diagnoses also had the largest number of infections averted. (DOCX) [file pone.0142576.s003.docx]
