# Supplementary material for: Partner Notification for Reduction of HIV-1 Transmission and Related Costs among Men Who Have Sex with Men: A Mathematical Modeling Study
Source: PLoS One. 2015 Nov 10;10(11):e0142576. doi: 10.1371/journal.pone.0142576 (PMC4640527; doi:10.1371/journal.pone.0142576)
Supplement: S1 Table — (DOCX) [file pone.0142576.s004.docx]

| Parameter used to accept simulations | Values Accepted | Source |
| --- | --- | --- |
| MSM population 2013-2015 | 164,000-190,000 | [[1](#_ENREF_1),[2](#_ENREF_2)] |
| MSM population 2035 | <200,000* | [[1](#_ENREF_1),[2](#_ENREF_2)] |
| Number HIV diagnoses in 2008 | 800 (range 500-1100) | [[3](#_ENREF_3)] |
| Number HIV diagnoses in 2012 | 700 (range 400-1000) | [[3](#_ENREF_3)] |
| Percent decrease in new HIV diagnoses from 2008-2012 | 12.5% (range 0%-25% decrease) | [[3](#_ENREF_3)] |
| Percentage diagnosed with CD4 count <200 cells/µl in 2012 | 20% (range 15%-25%) | [[3](#_ENREF_3)] |
| Percentage diagnosed with CD4 count 200-350 cells/µl in 2012 | 17% (range 12%-22%) | [[3](#_ENREF_3)] |

*To represent modest or stable population growth, as is observed in the Netherlands

1. Marcus U, Hickson F, Weatherburn P, Schmidt AJ, Network E (2013) Estimating the size of the MSM populations for 38 European countries by calculating the survey-surveillance discrepancies (SSD) between self-reported new HIV diagnoses from the European MSM internet survey (EMIS) and surveillance-reported HIV diagnoses among MSM in 2009. Bmc Public Health 13: 919.

2. The World Bank (2014) Netherlands: World Development Indicators.

3. van Sighem A, Gras L, Kesselring A, Smit C, Engelhard E, et al. (2013) Monitoring Report 2013: Human Immunodeficiency Virus (HIV) Infection in the Netherlands. Amsterdam: Stichting HIV Monitoring (SHM).
