# Supplementary material for: Partner Notification for Reduction of HIV-1 Transmission and Related Costs among Men Who Have Sex with Men: A Mathematical Modeling Study
Source: PLoS One. 2015 Nov 10;10(11):e0142576. doi: 10.1371/journal.pone.0142576 (PMC4640527; doi:10.1371/journal.pone.0142576)
Supplement: S2 Table — (DOCX) [file pone.0142576.s005.docx]

| **Status** | **Utility Weight*** |
| --- | --- |
| Susceptible | 1.0 |
| CD4 cell count >350 cells/µl | 0.94 |
| CD4 cell count 200-350 cells/µl | 0.82 |
| Infected AIDS stage | 0.7 |
| Infected on treatment | 0.94 |
| *Weights based on a pooled analysis by Tengs and Lin (2002) [[1](#_ENREF_1)] | |

1. Tengs TO, Lin TH (2002) A meta-analysis of utility estimates for HIV/AIDS. Med Decis Making 22: 475-481.
