# Supplementary material for: Partner Notification for Reduction of HIV-1 Transmission and Related Costs among Men Who Have Sex with Men: A Mathematical Modeling Study
Source: PLoS One. 2015 Nov 10;10(11):e0142576. doi: 10.1371/journal.pone.0142576 (PMC4640527; doi:10.1371/journal.pone.0142576)
Supplement: S1 Text — (DOCX) [file pone.0142576.s006.docx]

The model is seeded in 1975 with one infected individual. The state variables and HIV transmission equations for the model are shown below. There are four activity classes *i* based on the partner acquisition rate change: class 1 in which individuals have 210-556 partners per year, class 2 with 11-90 partners, class 3 with 1-10 and class 4 with 0.4-2.

The model included six HIV infection stages *k*: stage 1 is the acute stage. Stage 2, 3, and 4 are the chronic stages, divided up by CD4 cell count (stage 2 >500 cells/µl; stage 3 350-500 cells/µl; and stage 4 200-350 cells/µl). Stage 5 is the pre-final AIDS stage in which individuals have limited sexual activity. Class 6 is the final AIDS stage in which patients do not have any sexual intercourse [[1](#_ENREF_1)]. Infected and not yet treated individuals can be in class *U,* undiagnosed, *N*, notified and not yet tested, or *T*, tested.

During treatment, the model includes two infection stages *l*: class 1 are individuals who were in the recent or chronic stage before start of treatment, class 2 are patients who were in one of the AIDS stage before antiretroviral therapy was initiated.

Patients progress through three treatment stages *m*: The first two treatment stages occur, respectively, during the first three months (stage 1) and months four to six after start of treatment (stage 2). Patients receiving antiretrovirals for more than six months are in stage 3.

The population used is the estimated number of MSM living in the Netherlands, an estimated 140,000 in 1975 when the model was started, and 164,000-190,000 MSM in 2014 [[2](#_ENREF_2),[3](#_ENREF_3)].

**State variables**

$E_{i}$ = Entry rate susceptible individuals, i=1..4

$S_{i}$ = Susceptible individuals, i=1..4

$I_{i,k}^{U}$ = HIV infected individuals, unaware of their infection, i=1..4, k=1..6

$I_{i,k}^{N}$ = HIV infected individuals, notified to get tested from infected partner, i=1..4, k=1..6

$I_{i,k}^{T}$ = HIV infected individuals who tested positive for HIV, i=1..4, k=1..6

$I_{i,l,m}^{Rx}$ = Infected individuals receiving treatment, i=1..4, l=1..2, m=1..3

**Other variables**

$\lambda_{i}$ = U Force of infection, i=1..4

$N_{i}$ = Number of individuals in sexual activity class *i*, i=1..4

$\mu$ = Mortality general population

$\mu_{k}$ = Mortality untreated HIV infected patients in infection stage *k,* k=1..6

$\mu_{l,m}^{Rx}$ = Mortality treated patients in treatment stages *l* and *m,* l=1..3, m=1,2

$\gamma_{k}$ = HIV infection progression rate by stage k, k=1..6

$\tau_{k}$ = Proportion of patients tested for HIV in stage k, k=1..6

$RX_{k}$ = Proportion of patients starting treatment in progression stage k, k=1..6

$\rho$ = Proportion of patients that are linked into care

$\omega$ = Partner notification rate

$\varphi$ = Population growth rate

$\alpha$ = The reduction in infectiousness on people on treatment

**Ordinary Differential Equations**

(1) $S_{i}=S_{i}\left( \varphi\right)-S_{i}\sum_{k=1}^{6} \lambda_{i}-S_{i}\mu$

(2) $I_{i,1}^{U}=S_{i}\sum_{k=1}^{6} \lambda_{i}-I_{i,1}^{U}(\mu_{1}+\gamma_{1}+\tau_{1}^{U}+\omega$)

(3) $I_{i,k}^{U}={\gamma_{k-1}I}_{i,k-1}^{U}-I_{i,k}^{U}\left( \mu_{k}+\gamma_{k}+\tau_{k}^{U}+\omega\right)$ k=2..5

(4) $I_{i,6}^{U}={\gamma_{5}I}_{i,5}^{U}-I_{i,6}^{U}\left( \mu_{6}+\tau_{6}^{U}+\omega\right)$

(5) $I_{i,1}^{N}={\omega I}_{i,1}^{U}-I_{i,1}^{N}(\mu_{1}+\gamma_{1}+\tau_{1}^{N})$

(6) $I_{i,k}^{N}={\omega I}_{i,k}^{U}+{\gamma_{k-1}I}_{i,k-1}^{N}-I_{i,k}^{N}(\mu_{k}+\gamma_{k}+\tau_{k}^{N})$ k=2..5

(7) $I_{i,6}^{N}={\omega I}_{i,6}^{U}+{\gamma_{5}I}_{i,5}^{N}-I_{i,6}^{N}(\mu_{6}+\tau_{6}^{N})$

(8) $I_{i,1}^{T}=\tau_{1}^{U}I_{i,1}^{U}+\tau_{1}^{N}I_{i,1}^{N}-I_{i,1}^{T}(\mu_{1}+\gamma_{1}+{\rho RX}_{1})$

(9) $I_{i,k}^{T}=\tau_{k}^{U}I_{i,k}^{U}+\tau_{k}^{N}I_{i,k}^{N}+{\gamma_{k-1}I}_{i,k-1}^{T}-I_{i,k}^{T}(\mu_{k}+\gamma_{k}+{\rho RX}_{k})$ k=2..5

(10) $I_{i,6}^{T}=\tau_{6}^{U}I_{i,6}^{U}+\tau_{6}^{N}I_{i,6}^{N}+{\gamma_{5}I}_{i,5}^{T}-I_{i,6}^{T}(\mu_{6}+{\rho RX}_{6})$

(11) $I_{i,1,1}^{Rx}=\rho\left( {{RX}_{1}I}_{i,1}^{T}+{{RX}_{2}I}_{i,2}^{T}+{{RX}_{3}I}_{i,3}^{T}+{{RX}_{4}I}_{i,4}^{T} \right)-I_{i,1,1}^{Rx}(4+\mu_{1,1}^{Rx})$

(12) $I_{i,2,1}^{Rx}=\rho\left( {{RX}_{5}I}_{i,5}^{T}+{{RX}_{6}I}_{i,6}^{T} \right)-I_{i,2,1}^{Rx}(4+\mu_{2,1}^{Rx})$

(13) $I_{i,l,2}^{Rx}=4I_{i,l,1}^{Rx}-I_{i,l,2}^{Rx}(4+\mu_{l,2}^{Rx})$

(14) $I_{i,l,3}^{Rx}=4I_{i,l,2}^{Rx}-I_{i,l,3}^{Rx}(\mu_{l,3}^{Rx})$

**Force of infection**

The equation for the force of infection includes a mixing matrix $M_{i,j}$ for infected individuals, with a different infectiousness for each stage of infection, $\beta_{k}$. The elements of this matrix are *i,j* and represent the probability that an individual with *i* new partnerships per year will form a new partnership with a member who has *j* new partners. The rate at which the sexual partner changes for individuals in each sexual activity group *i* is expressed as $c_{i}$. The values of the matrix depend on the degree of mixing ε. This degree can be fully assortative (ε=1), where partnerships are only formed within the same activity class. Or fully random (ε=0), where partnerships are randomly formed between different activity classes [[4](#_ENREF_4)].

(15)$M_{i,j}=\varepsilon\delta+\frac{(1-\varepsilon)c_{j}N_{j}}{\sum_{i=1}^{4} c_{i}N_{i}}$

Where δ = 1 when *i = j*, and δ = 0 when *i ≠ j*.

(16) $\lambda_{i}=\lambda_{i}^{U}+\lambda_{i}^{N}+\lambda_{i}^{T}+\lambda_{i}^{Rx}$

(17) $\lambda_{i}^{U}= c_{i}\sum_{j=1}^{4} \frac{M_{i,j}}{N_{j}}(\sum_{k=1}^{6} I_{i,k}^{U}\beta_{k})$

(18)$\lambda_{i}^{N}= c_{i}\sum_{j=1}^{4} \frac{M_{i,j}}{N_{j}}(\sum_{k=1}^{6} I_{i,k}^{N}\beta_{k})$

(19)$\lambda_{i}^{T}= c_{i}\sum_{j=1}^{4} \frac{M_{i,j}}{N_{j}}(\sum_{k=1}^{6} I_{i,k}^{T}\beta_{k})$

(20)$\lambda_{i}^{Rx}= c_{i}\sum_{j=1}^{4} \frac{M_{i,j}}{N_{j}}(\sum_{l=1}^{2} {(\sum_{m=1}^{3} \alpha I}_{i,l,m}^{Rx}\beta_{2}))$

In which $\lambda_{i}$ is the force of infection due to contact with an infected person. Similarly, $\lambda_{i}^{N}$ is the force of infection due to contact with a person who has been notified by a known infected individual and has not yet tested, and $\lambda_{i}^{T}$is the force of infection due to contact with a person that has been tested positive for HIV, on treatment or not. $\lambda_{i}^{Rx}$ is the force of infection due to contact with a person who is on treatment, and has a reduced infectiousness, $\alpha$.

1. Hollingsworth TD, Anderson RM, Fraser C (2008) HIV-1 transmission, by stage of infection. J Infect Dis 198: 687-693.

2. Marcus U, Hickson F, Weatherburn P, Schmidt AJ, Network E (2013) Estimating the size of the MSM populations for 38 European countries by calculating the survey-surveillance discrepancies (SSD) between self-reported new HIV diagnoses from the European MSM internet survey (EMIS) and surveillance-reported HIV diagnoses among MSM in 2009. Bmc Public Health 13: 919.

3. The World Bank (2014) Netherlands: World Development Indicators.

4. Garnett GP, Anderson RM (1993) Factors controlling the spread of HIV in heterosexual communities in developing countries: patterns of mixing between different age and sexual activity classes. Philos Trans R Soc Lond B Biol Sci 342: 137-159.
